# Mechanism confirmation of organofunctional silanes modified sodium silicate/polyurethane composites for remarkably enhanced mechanical properties

**DOI:** 10.1038/s41598-021-88893-2

**Published:** 2021-04-30

**Authors:** Yuntao Liang, Ao Gao, Yong Sun, Fuchao Tian, Weili Sun, Wei Lu, Zhenglong He

**Affiliations:** 1 State Key Laboratory of Coal Mine Safety Technology, China Coal Technology & Engineering Group Shenyang Research Institute, Shenfu Demonstration Zone, 113122 China; 2 State Key Laboratory of Mining Disaster Prevention and Control Co-Founded by Shandong Province and Ministry of Science and Technology, Shandong University of Science and Technology, Qingdao, 266590 China; 3 College of Safety and Environmental Engineering, Shandong University of Science and Technology, Qingdao, 266590 China; 4 National Demonstration Center for Experimental Mining Engineering Education, Shandong University of Science and Technology, Qingdao, 266590 China

**Keywords:** Chemistry, Materials science

## Abstract

Hybrid reinforced sodium silicate/polyurethane (SS/PU) composites mainly derived from low-cost SS and polyisocyanate are produced by a one-step method based on the addition of 3-chloropropyltrimethoxysilane (CTS). The wettability of SS on PU substrate surface is much improved as CTS content increases from 0.0 to 3.5 wt%. Furthermore, with 2.5 wt% of CTS optimal addition, the fracture surface morphology and elemental composition of the resulting SS/PU composites are characterized, as well as mechanical properties, chemical structure and thermal properties. The results indicate that the CTS forms multiple physical and chemical interactions with the SS/PU composites to induce an optimized organic–inorganic hybrid network structure thus achieving simultaneous improvement of compressive strength, flexural strength, flexural modulus and fracture toughness of the SS/PU composites, with the improvement of 12.9%, 6.6%, 17.5% and 9.7%, respectively. Moreover, a reasonable mechanism explanation for CTS modified SS/PU composites is confirmed. Additionally, the high interface areas of the organic–inorganic phase and the active crosslinking effect of the CTS are the main factors to determine the curing process of the SS/PU composites.

## Introduction

Polyurethane have various applications including the mining industry^1–4^, building foundation reinforcement^5–7^, road maintenance^8–12^, textiles^13^ and other areas^14^, because of its excellent characteristics in terms of efficient performance and easy operation. However, it is found that the high cost, high heat release and flammability of polyurethane production limit its application. Especially, these disadvantages are fatal and prohibit in the field of coal mining. To overcome these disadvantages, inorganic modification is a promising strategy in improving the performance of the composites^15,16^. Hybrid organic/inorganic composites made from polyisocyanate and sodium silicate have low-cost, good permeability, low heat release and fire resistance, but have poor mechanical properties due to the interface incompatibility between organic and inorganic phase^17^. Various new interface modification means^18,19^ and additives have been used as an emulsifier for the improvement of the SS/PU composites performance, such as phosphate^20^, vinyl ester^21^, melamine formaldehyde^22^, epoxy^23^. Although several additives have shown effective mechanical reinforcements in SS/PU composites, it is difficult to simultaneously improve the compressive strength, flexural strength, flexural modulus and the fracture toughness of the SS/PU composites. Later, He^2^ and Kopietz^1^ proposed that the mechanical properties (e.g. compressive, fracture, flexural and dynamic mechanical properties) of SS/PU composites could be dramatically improved by using the organofunctional silanes. Although it is confirmed that the particle dispersion and cavitation can promote the strength and toughness of the SS/PU composites, the mechanism of the organofunctional silanes acting on the SS/PU composites remains unclear. Moreover, previous studies have shown a debonding cavitation between organic phase and inorganic phase due to the volume shrinkage of the inorganic phase during the hydrogel-xerogel transition. The introduction of the organofunctional silanes in the SS/PU composites can not promote the interfacial bonding between the inorganic phase and the organic phase. Therefore, understanding the behavior mechanism of the organofunctional silanes in the SS/PU composites to achieve SS/PU composites with good reinforcement performances is highly desirable.

Herein, a highly mechanical performance SS/PU composites from a facile in-situ polycondensation between SS and polyisocyanate are achieved by using the organofunctional silanes 3-chloropropyltrimethoxysilane (CTS). The effect of the CTS on the wettability of the SS and the compressive strength of the SS/PU composites are investigated. Meantime, it also evaluates the flexural and fracture properties of the SS/PU composites with the optimum dosage of CTS. Furthermore, according to the results and analyses of Scanning electron microscopy (SEM), Energy dispersive spectrometry (EDS), Fourier transform infrared (FTIR), Diffuse reflectance infrared Fourier transform spectroscopy (DRIFTS), Thermogravimetric analysis (TGA) and Differential scanning calorimetry (DSC) measurements, the curing mechanism of the SS/PU composites by introducing CTS is discussed.

## Experiments

### Materials

The polyisocyanate (PM-200, isocyanate content: 30.5–32 wt%) is kindly supplied by Yantai Wanhua Polyurethanes Co., Ltd. The polyether polyol with molecular weights of 2000 (GE-220, Hydroxyl number: 54.5–57.5 mg KOH/g) and chlorinated paraffin-52 are purchased from Shanghai Gaoqiao Petrochemical Co., Ltd. Sodium silicate (SS) is purchased from Shandong Hongquan Chemical try Co., Ltd. 3-chloropropyltrimethoxysilane (CTS, purity: 98%) and cyclohexylamine (purity: 98%) are obtained from Shanghai Aladdin Biochemical Technology Co., Ltd.

### Sample preparation

SS/PU composites are prepared via a conventional mixing and curing procedure of two components. Firstly, 30 g of SS and different amount (0–1.05 g) of CTS are placed in a 500 mL plastic cup and homogenized at 300 rpm for 30 min to promote the uniform distribution and hydrolysis of CTS in SS. Then, 0.15 g of cyclohexylamine as a catalyst is added and mixed for 4 min at 500 rpm. The obtained emulsion is defined as the inorganic component. A certain amount of the organic component including PM-200(27 g), chlorinated paraffin-52 (3 g) and GE-220 (3 g) are added into the inorganic component and homogenized again for 1 min at 300 rpm. After mixing process, different dimensions of SS/PU composites are cast and cured in different steel molds at ambient condition, such as the compression test specimens (50 × 100 mm^2^, diameter × height), the flexural test specimens (60 × 10 × 3 mm^3^, length × width × height) and the fracture toughness test specimens (35 × 35 × 3 mm^3^, length × width × thickness).

### Sample characterization

The wettability alteration behavior for the SS/CTS system is characterized by the contact angle between the modified SS and the organic component. A droplet of SS with different CTS contents is placed on the cured organic component surface. Images of the equilibrium contact angle of the mixed SS/CTS droplet are recorded by the SL200B machine (KINO Industry Co., Ltd., USA). The mechanical tests of the compressive, flexural and fracture properties of the SS/PU composites are performed on an electron omnipotence experiment machine (SANS-CMT6503, Shenzhen Sans Testing Machine Co., China) at ambient conditions according to GB/T 1041-1992, EN 63 and ISO-13586-1, respectively. The testing results are reported as the average of at least three measurements. The fracture surface morphology and the elemental composition of the SS/PU composites are analyzed by a scanning electron microscope (SEM, 2800B, KYKY, China) with an energy dispersive spectrometer (EDS, QX200, Bruker, Germany). Bruker Tensor 27 FTIR Spectrometer (Bruker, Germany) is used for the quantitative analysis^24^ of isocyanate (NCO) peak intensity in the SS/PU composites under different curing time. Diffuse reflectance infrared Fourier transform spectroscopy (DRIFTS) is recorded for the fully cured modified and non-modified SS/PU composites by using a Bruker Tensor 27 instrument equipped with a MCT detector. Thermogravimetric analysis (TGA) is performed using a PerkinElmer STA6000 thermal analyzer at 10 °C·min^−1^ heating rate from 30 °C to 600 °C under a nitrogen atmosphere with a flow rate of 30 mL·min^−1^. Differential scanning calorimetry (DSC) analysis is conducted on a Mettler-Toledo DSC823e system to study the thermal properties of the modified and non-modified SS/PU composites under a nitrogen atmosphere at a heating rate of 10 °C·min^−1^ within the temperature’s range from 30 to 250 °C.

## Results and discussion

### Synthesis mechanism of CTS modified SS/PU composites

**Figure 1:**
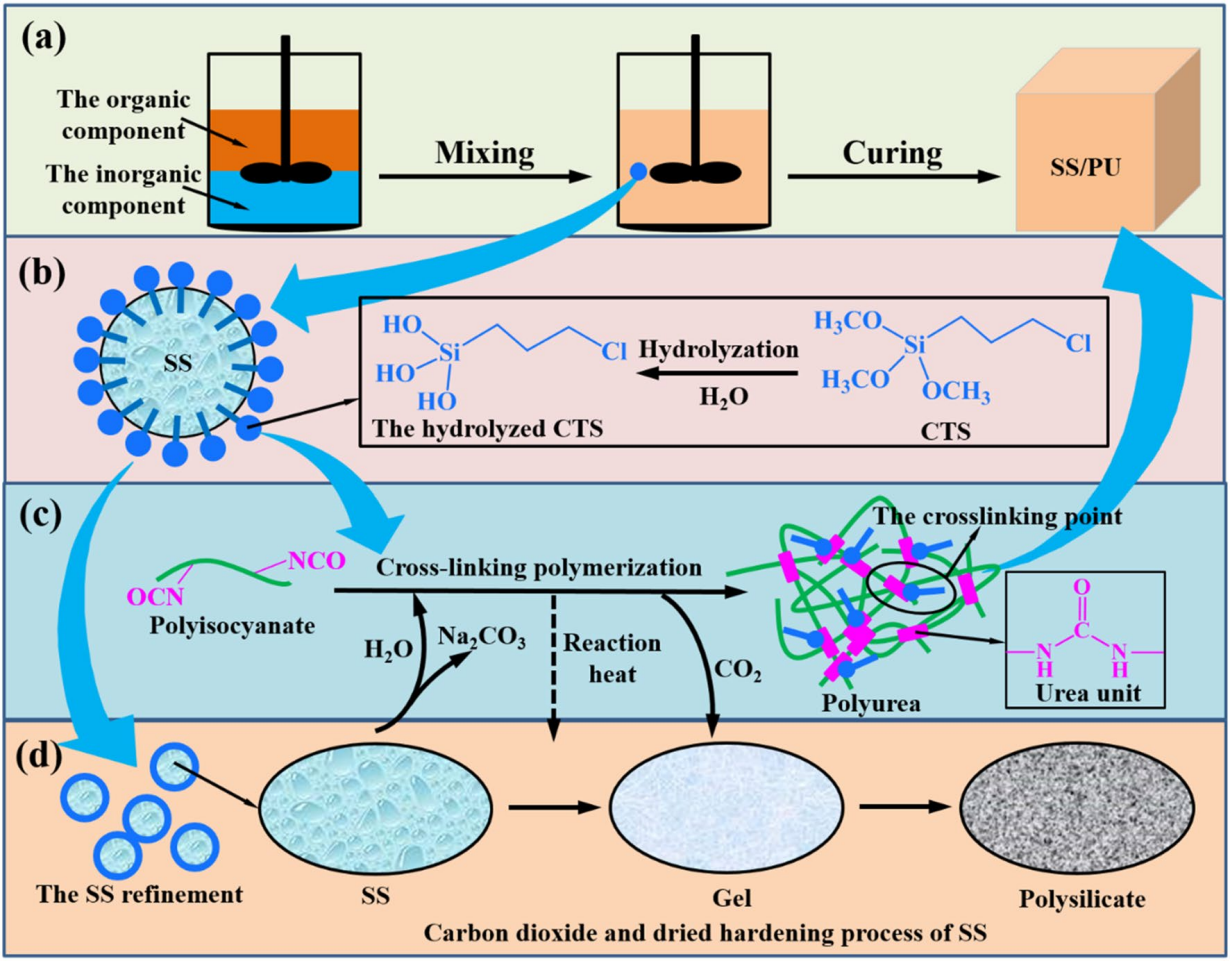
The proposed mechanism for the synthesis of CTS modified SS/PU composites.

Figure 1 schematically shows the primary chemical reactions route that occurs during the SS/PU composites preparation and the CTS effect. The organic component and the modified SS are mixed by using a two-blade paddle mixer. The achieved homogeneous mixed slurry is poured into the mold and cured until theexpected SS/PU composites are achieved (see Fig. 1a). During the process of mixing, the hydrolyzed CTS is oriented at the interface between the organic phase and the inorganic phase, thus forming a smaller and more uniform inorganic dispersion system by the reduction of the organic–inorganic interfacial tension (see Fig. 1b,d). This benefits the acceleration of the curing process and the better dispersion of the stress. Thus, the mechanical properties of the composites are further improved. During the following curing of SS/PU composites, multifunctional polyisocyanate is reacted with the water from the SS, leading to a highly cross-linking network of formed polyurea by urea units in the organic phase (see Fig. 1c)^25^. More water consumption produces more carbon dioxide into SS, which in turn produces more sodium carbonate, leading to gelation of the SS in the inorganic phase^23^. Water continuously enters the organic phase from the inorganic phase, while the diffusion path of the produced carbon dioxide is opposite^26^. The directional mass transfer of water and carbon dioxide together realizes the hardening of the SS to the polysilicate and the cross-linking polymerization of the organic phase, and eventually produces the solidification of of the organic phase and the inorganic phase^2^. Moreover, a large amount of reaction heat from the amine-isocyanate chemistry will also accelerate this curing process. In addition, the lipophilic group can make part of CTS get into the organic phase well-distributed. Subsequently, the reactive lipophilic groups of the CTS as hard segment will bind with the cross-linking point (e.g. the NCO and urea groups) of the organic phase, forming a chemical bond. Therefore, it is precise because of multiple physical and chemical interactions between the CTS and the SS/PU composites that the SS/PU composites have the outstanding mechanical properties.

### Contact angle analysis of the modified SS

**Figure 2:**
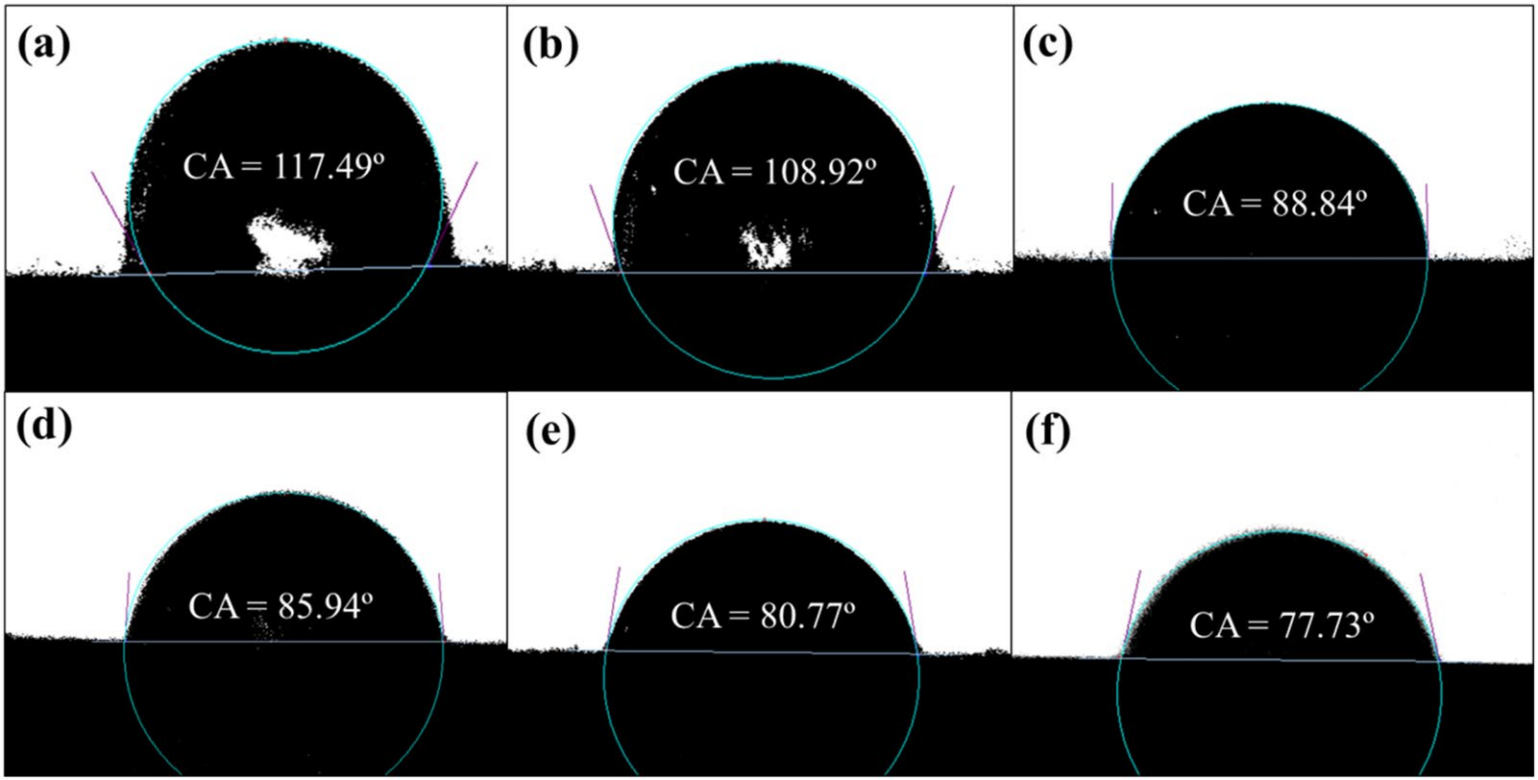
Contact angle images of SS with different CTS content: (**a**) 0.0 wt%, (**b**) 1.5 wt%, (**c**) 2.0 wt%, (**d**) 2.5 wt%, (**e**) 3.0 wt%, (**f**) 3.5 wt%.

To achieve a high mechanical performance of the composites, interface properties between the organic and the inorganic phases need to be improved. Figure 2 shows the static contact angle of the modified SS on the cured organic component surface. Pure SS presents a contact angle of 117.49°, whereas by modifying it is gradually reduced from 117.49° to 77.73°. An improvement of more than 33% is observed in wettability of SS on the cured organic component surface. It is probably ascribed to CTS modification decreasing the surface energy and hydrophilicity index of the SS, so SS becames much more hydrophobic and stronger affinity toward the organic phase^27^. This improved wetting property is beneficial to the refinement and uniform distribution of the SS in PU matrix and improves the strength of the SS/PU composites.

### Mechanical properties of SS/PU composites

**Figure 3:**
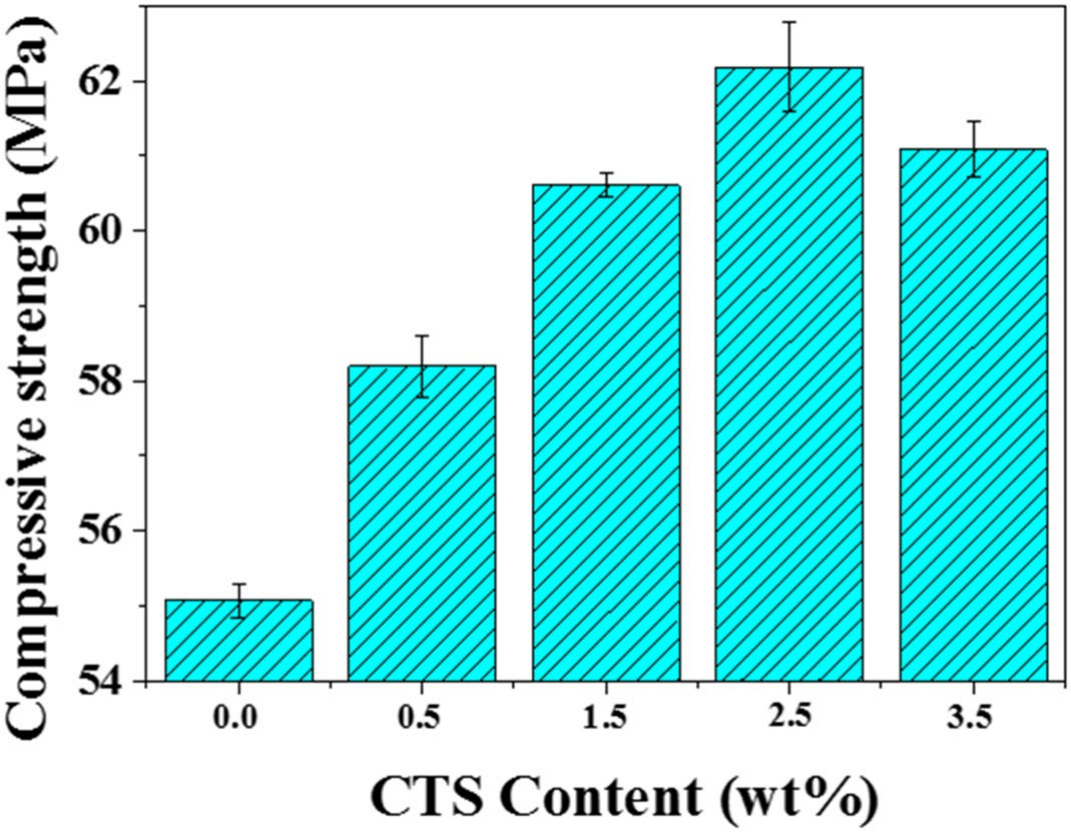
Compressive strength of the SS/PU composites with different CTS contents.

Figure 3 shows that the compressive strength obtained form the SS/PU composites with the CTS content of 0.0 wt%, 0.5 wt%, 1.5 wt%, 2.5 wt% and 3.5 wt%. The CTS is observed to enhance the compressive strength of the SS/PU composites significantly. Excellent wettability allows maximum inorganic phase refinement. Accordingly, the curing process of the SS/PU composites becomes faster and more complete. Therefore, the compressive strength of the SS/PU composites varies from 55.07 to 62.19 MPa with the increase of the CTS content from 0.0 wt% to 2.5 wt%. As CTS content is greater than 2.5 wt%, a peak is reached for the SS/PU composites, showing a slight decrease in compressive strength thereafter. This is probably due to the arising interface saturation with the CTS in the SS/PU composites. The outcome therefore indicates that the interface saturation between the inorganic phase and organic phase may start as early as the 2.5 wt% CTS content range, which is adequate to reach high compressive performance.

**Figure 4:**
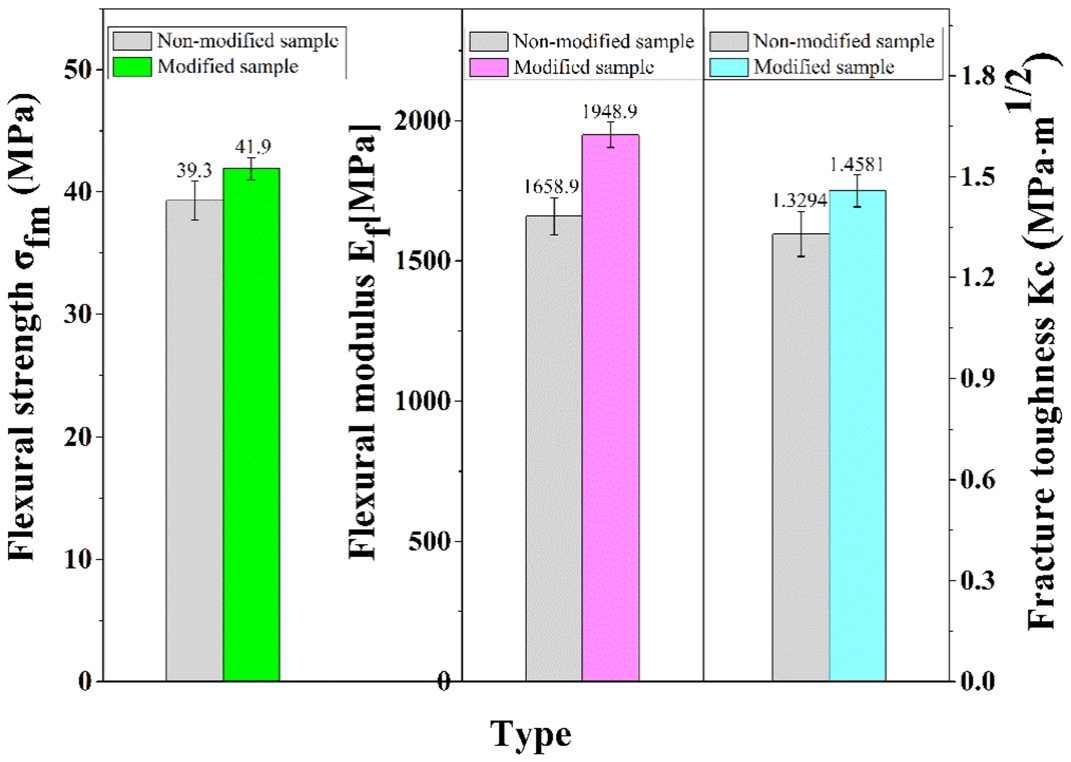
Flexural strength, flexural modulus and fracture toughness of the non-modified and modified SS/PU composites.

To further understand the mechanical properties of the SS/PU composites, the flexural strength, flexural modulus and fracture toughness of the SS/PU composites with 2.5% of CTS optimal addition and without CTS are examined respectively, as shown in Fig. 4. From Fig. 4, it is observed that 2.5 wt% of the CTS appropriately improves the average flexural strength of the SS/PU composites from 39.3 MPa to 41.9 MPa. However, the 17.5% and 9.7% enhancement in flexural modulus and fracture toughness are achieved from 2.5 wt% CTS modified the SS/PU composites, respectively. This may be attributed to the active cross-linking effect of the CTS. The CTS can enter and form strong chemical interactions with the organic phase of the SS/PU composites and further strengthen the interior cross-linking structure of the organic phase, thus facilitating the improvement of strength and toughness for the SS/PU composites. Therefore, 2.5 wt% of CTS will be used as the designated amount of modified SS/PU composites. The obtained SS/PU composites will be used for the fracture surface morphology, elemental composition, chemical structure and thermal properties analysis.

### SEM–EDS analysis of SS/PU composites

**Figure 5:**
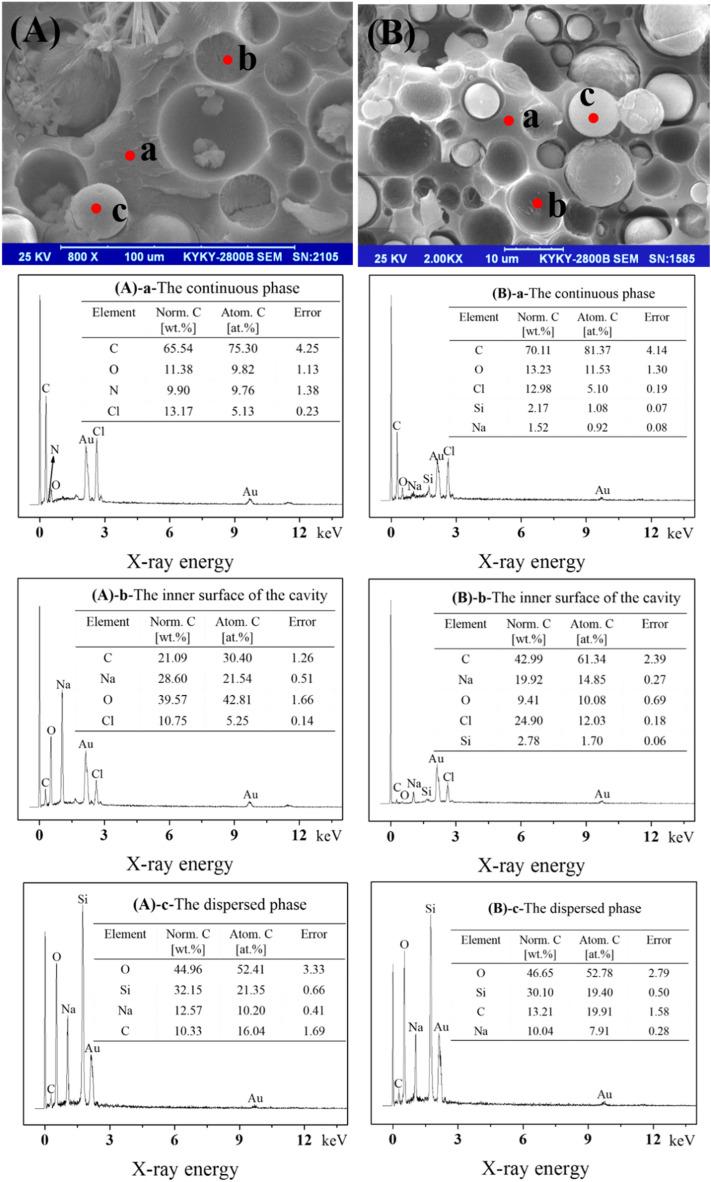
SEM images and EDS spectra of the fracture surface of (**A**) non-modified SS/PU and (**B**) modified SS/PU composites (**a**: the continuous phase, **b**: the inner surface of the cavity and **c**: the dispersed phase).

In order to understand constitutes of the SS/PU composites and further explore how the CTS produces physical and chemical interactions to enhance the mechanical performance of the SS/PU composites, SEM and EDS are used together to observe the fracture surface morphology and analyze the elemental composition and content in the SS/PU composites (see Fig. 5). The SEM images show the spherical dispersed phases embedded in the continuous phase. Moreover, the continuous phases exhibit many dome-shaped cavities throughout the fractured surface of the composites. It is obvious that there is an insufficient interfacial bond strength between the continuous phase and the dispersed phase. The EDS analyses show that the continuous phases consist of C (65.54 wt%), O (11.38 wt%), Cl (13.17 wt%) and C (70.11 wt%), O (13.23 wt%), Cl (12.98 wt%) in the non-modified and modified SS/PU composites, respectively. It reveals the continuous phase originated from the organic component. On the other hand, the main elements of the dispersed phase are Si (32.15 wt%), O (44.96 wt%), Na (12.57 wt%), C (10.33 wt%) and Si (30.10 wt%), O (46.65 wt%), Na (10.04 wt%), C (13.21 wt%) in the non-modified and modified SS/PU composites, respectively. The results therefore prove that the dispersed phase originates from the SS.

As shown in Fig. 5A(a),B(a), compared with the non-modified samples, 2.17 wt% of the Si element appears in the continuous phase of the modified sample. This phenomenon illustrates that the CTS will migrate into the organic phase in the modified SS/PU composites. Figure 5A(b) and B(b) show that the element content of C (42.99 wt%), Cl (24.90 wt%) and Si (2.78 wt%) on the cavity surface of the modified composites are much higher than that (C (21.09 wt%), Cl (10.75 wt%) and Si (trace))of the non-modified composites. This confirms that the CTS is widely distributed at the interface between the organic phase and the inorganic phase. Furthermore, it is found that C and Cl elements are greatly increased on the cavity inner surface of the modified SS/PU composites. This demonstrates that the introduction of CTS improves the interface compatibility and affinity between organic and inorganic phases. In addition, it can be clearly seen that the O (46.65 wt%) and C (13.21 wt%) content on the dispersed phase of the modified sample are higher than that (O (44.96 wt%) and C (10.33 wt%)) of the non-modified sample (see Fig. 5A(c),B(c)). This increment is primarily attributable to higher CO_2_ accumulation rates at the inorganic–organic phase interface caused by the rapid cross-linking polymerization of the organic phase in the modified SS/PU composites.

**Figure 6:**
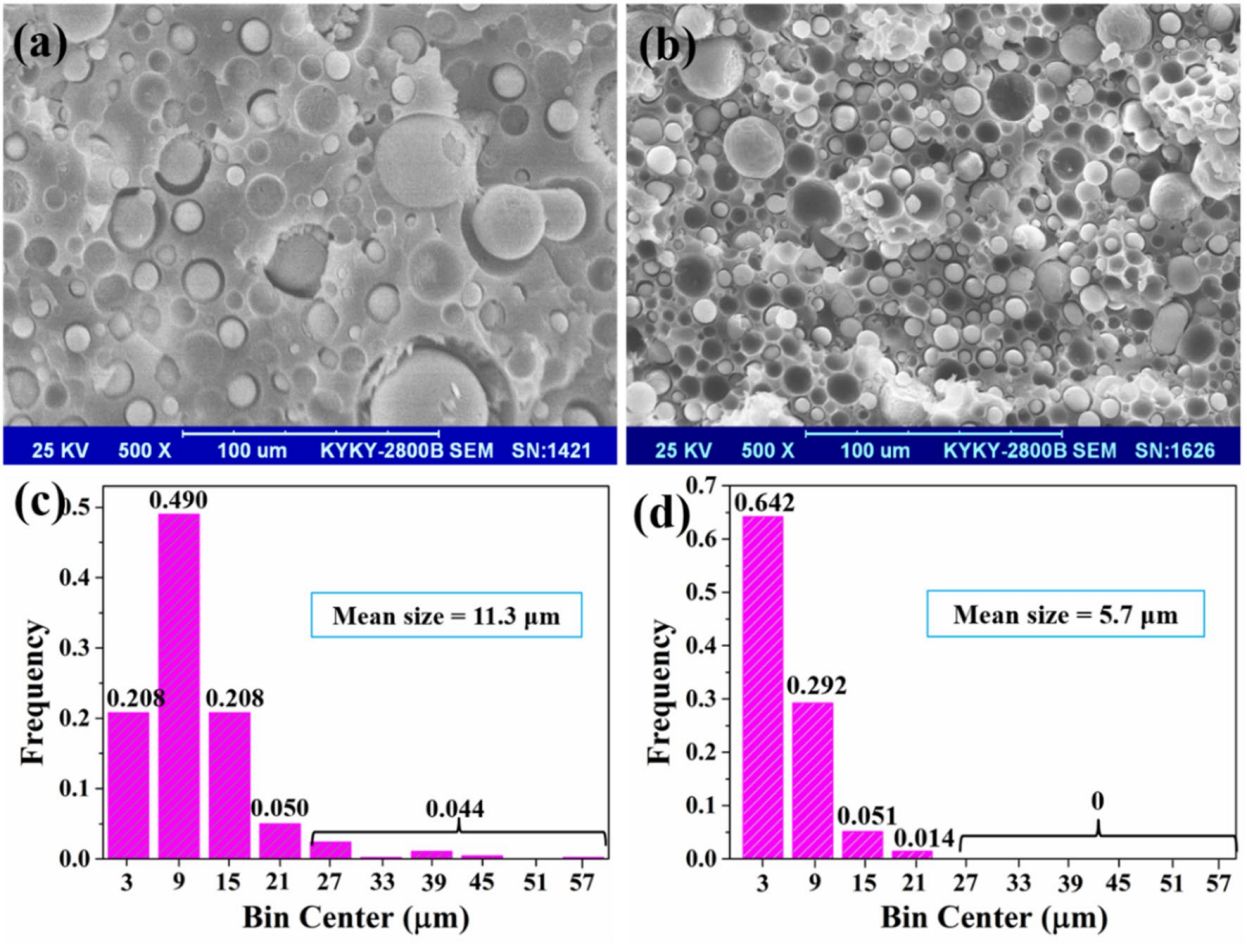
The cross-sectional SEM images of (**a**) non-modified and (**b**) modified SS/PU composites; and size distribution histograms of the polysilicate particles in (**c**) non-modified and (**d**) modified SS/PU composites.

In order to confirm the influence of CTS on the distribution and morphology of SS in the PU matrix. The cross-sectional SEM images of the non-modified and modified SS/PU composites are shown in Fig. 6a,b. The SEM images confirm that the polysilicate particles are individually dispersed in the PU matrix. Moreover, the SEM images show that the polysilicate particle size and dome-shaped cavities have become smaller and more uniform in the modified SS/PU composites due to the addition of CTS, which is responsible for the relatively high mechanical strength and toughness of SS/PU composites. In addition, Fig. 6c,d directly show the size distribution histograms of the polysilicate particles of the non-modified and modified samples. The size of polysilicate particle varies from 0 μm to 60 μm, and 0 μm to 24 μm for the non-modified and modified samples, respectively. Moreover, the mean size of polysilicate particle for the non-modified and modified samples are also reduced from 11.3 μm to 5.7 μm. The results demonstrate that the CTS has excellent emulsification ability. Additionally, 4.4% of the polysilicate particles with a scale between 24 μm and 60 μm are present in non-modified samples. Adding the size of polysilicate particles leads to larger cavities formation. These particles and cavities as defects can easily cause stress concentration, which deteriorates the mechanical properties of the SS/PU composites. The micro-structural analysis of SS/PU composites ensures the excellent emulsifying ability of the CTS, which is consistent with the results of mechanical performance testing.

### IR spectra of SS/PU composites

**Figure 7:**
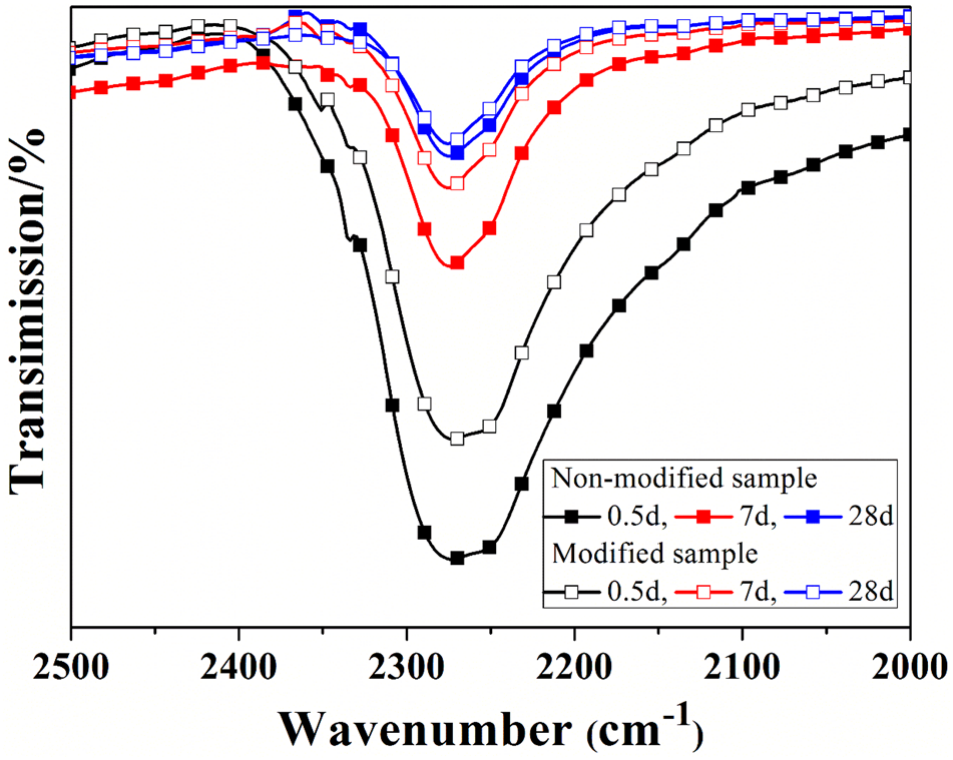
IR spectra of NCO group in the non-modified and modified SS/PU composites during the curing process.

The intensity of the NCO peak is a very important parameter, expressing the degree of curing of SS/PU composites. The strong absorption peak at 2270 cm^−1^ corresponds to the NCO group stretching in the SS/PU composites. As shown in Fig. 7, whether modified or not, the NCO peak intensity of the SS/PU composites gradually decreases as the the curing time increases from 0.5 to 28 days. This implies that NCO groups in the organic phase are gradually consumed by the diffused water from the inorganic phase during the curing process of the SS/PU composites. Moreover, by comparison, the NCO peak intensity of the modified SS/PU composites is weaker than that of the non-modified SS/PU composites under the same curing time. This difference indicates that the curing process of the SS/PU composites can be accelerated by the addition of CTS due to the intensity of the peak decreasing progressively as the curing rate increases. In addition, in comparison with the non-modified SS/PU composites, the absorption peak of NCO groups exhibited by the modified SS/PU composites decreases after fully curing. This is due to the fact that the CTS enters the organic phase as the additional cross-linking points form chemical interactions with NCO groups in the organic phase.

**Figure 8:**
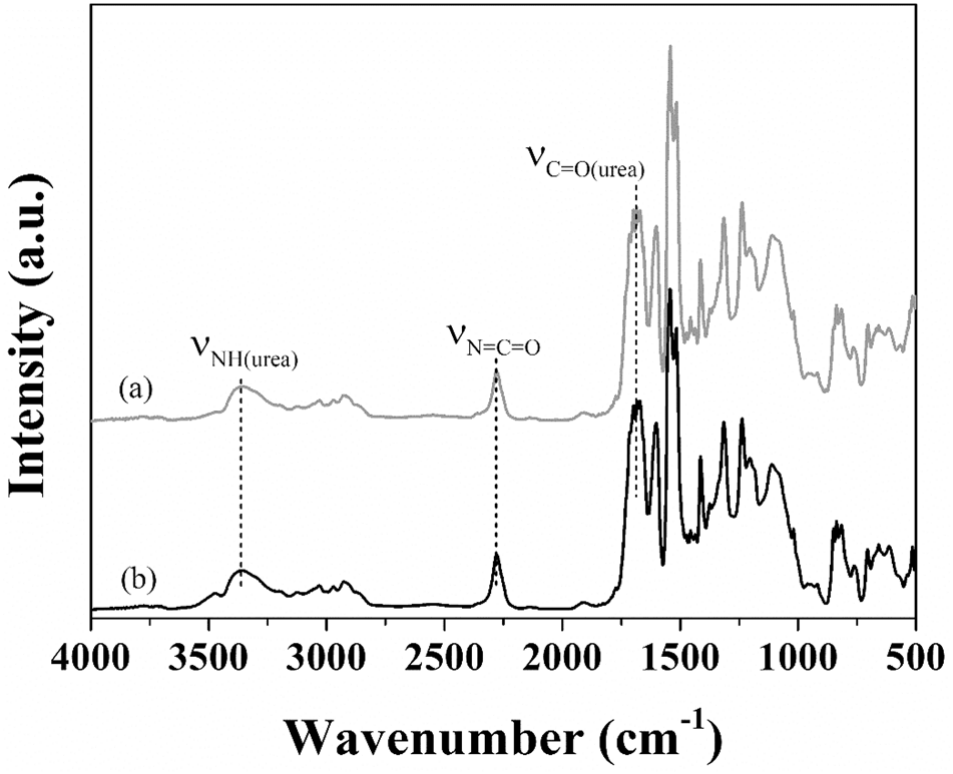
DRIFTS spectra of (**a**) modified and (**b**) non-modified SS/PU composites.

The DRIFTS spectrum obtained form the modified SS/PU composites is compared with those form the non-modified SS/PU composites in Fig. 8, which shows that the nearly identical spectrum curves of modified and non-modified SS/PU composites. This indicates that the addition of CTS has no obvious effect on the change of the chemical structure of the SS/PU composites. The absorption peaks at 3280–3360 cm^−1^ and 1680 cm^-1^ might contribute to amide N–H stretching peak and the urea carbonyl groups (C=O)^28–30^, which is the essential structures for polyurea from the reaction between NCO groups and water molecules. However, the characteristic peak of NCO at 2267 cm^-1^ is still observed, indicating that there might be a small amount of residual NCO in the SS/PU composites after fully curing.

### Thermo gravimetric analysis of SS/PU composites

**Figure 9:**
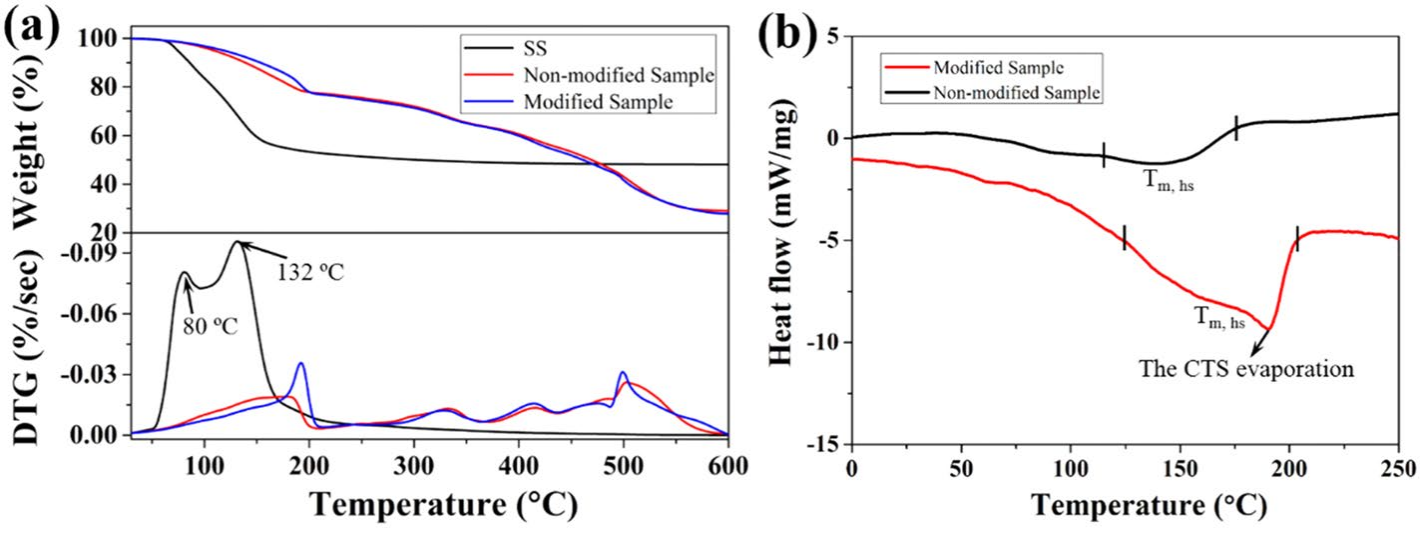
(**a**) TGA-DTG and (**b**) DSC curves of SS and SS/PU composites before and after modification.

In order to fully evaluate the effect of CTS on thermal properties of SS/PU composites, we investigate the course of thermal decomposition of SS and SS/PU composites before and after modification (see Fig. 9a). In the temperature range from 30to 250 °C the free water and the crystallization water are lost from the SS, with maximum rate at 80 °C and 132 °C, respectively. Similarly, the DTG peaks of SS/PU composites within 50–250 °C are due to elimination of water and a small amount of low molecular weight organics release. Between 50 and 178 °C, a slight reduction in degradation rate for modified SS/PU composites compares to non-modified SS/PU composites. It results from the fact the dispersion of SS particles in the PU matrix is higher and finer than that of non-modified SS/PU composites, therefore, the water from the SS can be more easily consumed. This is also the reason for the high thermal stability of the modified composite material between 30 °C and 198 °C. Moreover, a strong degradation rate peak is detected between 178 °C and 220 °C, which should be linked with the evaporation of the CTS present at the inorganic–organic phase interface of the modified SS/PU composites. Besides, three other typical weight loss steps are observed: the first and second mass loss occurring at 330 °C and 412 °C can be attributed to the degradation of hard and soft segment, respectively^31^. The third weight loss in the range of 480–550 °C might be due to the degradation of the rest of the organic phase^32^. In this step, the weight loss in the temperature range of 490 °C-506 °C seems to be ascribed to the decomposition of the CTS^33^. Moreover, the decomposition temperature is notably higher than the boiling point (195 °C) of the CTS because the CTS forms strong chemical bonds with the organic phase.

Figure 9b displays the DSC thermograms of the non-modified and modified SS/PU composites. The DSC thermograms show that there is a complex structure in the SS/PU composites. It will not clearly discern the glass transition temperature (T_g_) of the SS/PU composites. This phenomenon is attributed to that Tg is superimposed to thermal evaporation and degradation^34^. In the non-modified and modified SS/PU composites, the broad endothermic peak at 120–210 °C is ascribed to the endothermic melting peak of hard segment (T_m, hs_). The addition of CTS has a notable effect on the T_m, hs_ value of the SS/PU composites, which can be observed from the incremental change in T_m, hs_ values. It mainly attributed to the increasing of hard segments contents and crosslinking density by the CTS as the active hard segment crosslinker chemical links with the organic phase. A strong endothermic peak at 190 °C superimposes to the T_m, hs_, and the result confirms that the evaporation of the CTS because this temperature is close to the boiling point of free CTS. It also indicates that partially CTS is not bonded with the dispersed or continuous phases in the SS/PU composite, which is in good agreement with the TGA results.

## Conclusions

Highly mechanical properties of SS/PU composites made from multifunctional polyisocyanate and low-cost SS are successfully realized by introducing the organofunctional silanes CTS via the simple mixing. The addition of the CTS realizes the refinement and uniform distribution of the inorganic phase, and the acceleration of the curing process in the SS/PU composites. Meanwhile, the CTS as an active hard segment crosslinker forms chemical interactions with isocyanate groups in the organic phase. These effects will be beneficial to produce an optimized organic–inorganic hybrid network structure, thereby achieving simultaneous improvement of compressive strength, flexural strength, flexural modulus and fracture toughness of the SS/PU composites. Moreover, the rapid curing of the SS/PU composites composed of the hardening of the SS and the intermolecular crosslinks of the PU monomers is achieved by the high organic–inorganic phase interface areas and the active cross-linking effect of the CTS. The resultant work will provide the possibility to prepare high-performance SS/PU composites in potential engineering application fields.
